# Neutrophil-to-Lymphocyte Ratio (NLR): A Predictor of Microvascular Complications in Type 2 Diabetes Mellitus

**DOI:** 10.7759/cureus.98079

**Published:** 2025-11-29

**Authors:** Sunil K Behera, Md Sabah Siddiqui, Jhasaketan Meher, Rohini R

**Affiliations:** 1 General Medicine, All India Institute of Medical Sciences, Raipur, Raipur, IND

**Keywords:** diabetes mellitus, microvascular complication, nephropathy, neuropathy, neutrophil-to-lymphocyte ratio, nlr, retinopathy

## Abstract

Introduction

Diabetes is a major public health challenge in India, and simple, low-cost tools are needed to detect early microvascular complications. We explored whether the neutrophil-to-lymphocyte ratio (NLR) can help predict such complications in people with type 2 diabetes.

Methods

We conducted a cross-sectional study in 2022-23, enrolling 150 patients aged 40-80 years with type 2 diabetes. Each participant underwent a detailed clinical evaluation, including blood sugar, HbA1c, NLR, and screening for diabetic neuropathy, nephropathy, and retinopathy.

Results

The mean age of participants was 58.9 ± 9.3 years. Comorbidities were present in 84% of participants (n=126), mainly dyslipidaemia (n=102, 68%) and hypertension (n=86, 57.3%). Almost half of the patients (n=73, 48.6%) had neuropathy, 42.6% (n=64) had nephropathy, and about one-quarter (n=39, 26%) had retinopathy. The mean NLR was higher in patients with microvascular complications than in those without (p < 0.001). The NLR correlated strongly with neuropathy (r = 0.72, p < 0.001) and moderately with nephropathy (r = 0.39, p < 0.001) and retinopathy (p < 0.001). A higher NLR was also associated with poor glycaemic control (HbA1c) (r = 0.41, p < 0.001) and central obesity (waist-to-hip ratio) (r = 0.31, p < 0.001). NLR showed high prediction for neuropathy (cut-off = 1.75, AUC = 0.926, sensitivity 93.2%, specificity 74%) and retinopathy (cut-off = 2.15, AUC = 0.942, sensitivity 94.9%, specificity 78.4%), but not for nephropathy.

Conclusion

Although the NLR correlated significantly with all microvascular complications in T2DM, it showed strong predictive ability only for neuropathy and retinopathy. Thus, it may serve as a cost-effective marker for predicting microvascular complications.

## Introduction

The increasing prevalence of diabetes mellitus (DM) in India is a serious public health concern [1]. It is a metabolic disorder characterised by symptomatic or asymptomatic hyperglycemia, with type 2 diabetes mellitus (T2DM) comprising nearly 90% of all cases, which leads to microvascular and macrovascular complications if left untreated [2]. The International Diabetes Federation (IDF) reports that nearly 537 million people across the globe (10.5% of the total population) are affected by diabetes, and this figure may rise to 783 million by 2045 [2]. Diabetes shows an urban-rural divide, affecting a greater proportion of people in urban areas (12.1%) compared to rural areas (8.3%). Out of 90 million South Asian diabetes cases, India contributes about 74.2 million, which is expected to reach 124.9 million by 2045 [2]. The rapid rise in diabetes and its related complications is a concern worldwide, including in India. Identifying a low-cost, easily available, and valid blood marker for detecting diabetes-induced organ injury is essential to timely implement preventive therapy and management of microvascular complications.

Evidence suggests that inflammation and endothelial dysfunction are among the factors for the development of insulin resistance, diabetes, and subsequent vascular complications [3]. Previous studies have shown that high neutrophil levels and low lymphocyte levels may be an indicator of neuropathy, nephropathy, and retinopathy in diabetes [4]. There are reports stating that the neutrophil-to-lymphocyte ratio (NLR) can serve as a predictor of outcomes in patients with myocardial infarction, heart failure, and stroke [5,6]. Data on the predictive value of the NLR for microvascular complications in Indian individuals with T2DM are limited. Therefore, the present study aimed to determine whether the NLR can serve as an indicator of diabetic microvascular complications, including retinopathy, neuropathy, and nephropathy.

## Materials and methods

Study design and setting

This was a single-centred cross-sectional observational study, conducted at a tertiary health care centre in central India from March 2022 to August 2023. Informed consent was obtained from all eligible participants. Only the consented participants were enrolled in the study if they satisfied the inclusion and exclusion criteria.

Study population

Patients aged 40-80 years with T2DM were included in the study. Individuals were excluded if they had type 1 diabetes, a recent infection within the past month, haematological malignancies, autoimmune disorders, end-stage renal disease, chronic liver disease, or any condition affecting urinary protein excretion, such as nephrotic syndrome, urolithiasis, or urinary tract infections.

Methods

Detailed demographic information, diabetes history and management, comorbidities, and addiction history were recorded. Anthropometric measurements were also obtained. Complete blood count (CBC), fasting blood sugar, postprandial blood sugar, glycated haemoglobin (HbA1c), urine albumin-to-creatinine ratio (ACR), and lipid profile were performed. The NLR was calculated by dividing the absolute neutrophil count by the lymphocyte count from a CBC report. Diabetic neuropathy was diagnosed based on a detailed history and neurological examination, including monofilament testing, pinprick sensation, vibration and joint position assessment, and evaluation of deep tendon reflexes. Diabetic retinopathy was assessed using fundus examination. Diabetic nephropathy was assessed using the urine ACR.

Statistical analysis

Statistical analysis was conducted using IBM SPSS Statistics for Windows, Version 22 (Released 2013; IBM Corp., Armonk, New York, United States). The Kolmogorov-Smirnov or Shapiro-Wilk test was used to assess whether continuous variables followed a normal distribution. Categorical variables were compared using Fisher's exact test or Pearson's chi-square test. When appropriate, the Mann-Whitney U test or independent samples t-test was applied to compare quantitative data. One-way ANOVA or the Kruskal-Wallis test, as applicable, was used to compare more than two means. The association between NLR and microvascular complications was tested using univariate analysis. Receiver operating characteristic (ROC) curve analysis was performed to evaluate the discriminatory ability of the NLR for microvascular complications. We considered a p-value of ≤ 0.05 as statistically significant.

## Results

This study included 150 participants. The overall age range was 40-80 years, and the mean age of all participants was 58.95 ± 9.3 years. Male participants comprised a higher proportion than female participants (n=91, 60.7% vs n=59, 39.3%). Among the participants, 64% (n=96) reported a family history of diabetes. A large proportion (n=126, 84%) had additional comorbidities, with dyslipidaemia (n=102, 68%) and hypertension (n=86, 57.3%) being the most common. The mean duration of diabetes was 8 ± 4.32 years. Participants with at least one microvascular complication had a mean duration of 8.42 ± 4.33 years, whereas those without complications had a mean duration of 7.47 ± 4.27 years. Most participants (n=62, 41.3%) were in the Obese I category (BMI 25-29.9), with an overall mean BMI of 24.77 ± 2.47 kg/m². The mean HbA1c of the study population was 7.59 ± 1.18%.

Diabetic neuropathy was the most frequently observed microvascular complication, affecting 48.6% (n=73) of participants, followed by nephropathy (42.6%, n=64) and retinopathy (26%, n=39). Triopathy (all three complications) was observed in 24% (n=36) of participants, while 44% (n=66) had no microvascular complications. Baseline characteristics of the participants are given in Table 1.

**Table 1:** Baseline characteristics of the participants (n = 150) BMI: Body mass index; HbA1c: glycated haemoglobin

Parameters	n (%) or Mean ± S.D.
Age (years)	58.95±9.3
Male: Female	91 (60.7%): 59 (39.3 %)
Duration of diabetes (years)	8 ± 4.32
Family history of diabetes	96 (64%)
Alcoholic	37 (24%)
Hypertension	86 (57.3%)
Hypothyroidism	48 (32%)
Coronary artery disease	25 (16.6%)
Dyslipidaemia	102 (68%)
BMI (kg/m²)	24.77 ± 2.47
Waist-hip ratio (Male)	0.89±0.1
Waist-hip ratio (Female)	0.87±0.1
HbA1c (%)	7.59±1.18
Neuropathy	73 (48.6%)
Nephropathy	64 (42.6%)
Retinopathy	39 (26%)
Triopathy	36 (24%)
No microvascular complications	66 (44%)

The mean NLR was 2.13 ± 0.84. For analytical purposes, the NLR was categorised into four arbitrary ranges: 0.8-1.70, 1.71-2.60, 2.61-4.0, and >4.0 (Table 2).

**Table 2:** NLR categories and their proportions (n = 150) NLR: Neutrophil-to-lymphocyte ratio

NLR	Frequency (n); Percentage (%)
0.8 – 1.70	61 (40.6%)
1.71 – 2.60	55 (36.6%)
​​​​​2.61 – 4.0	28 (18.6%)
> 4.0	6 (4.2%)

Most participants (n=61, 40.6%) had an NLR between 0.8 and 1.70. The NLR exhibited a moderate positive correlation with both HbA1c levels (r = 0.41, p < 0.001) and waist-hip ratio (WHR) (r = 0.31, p < 0.001).

**Figure 1:**
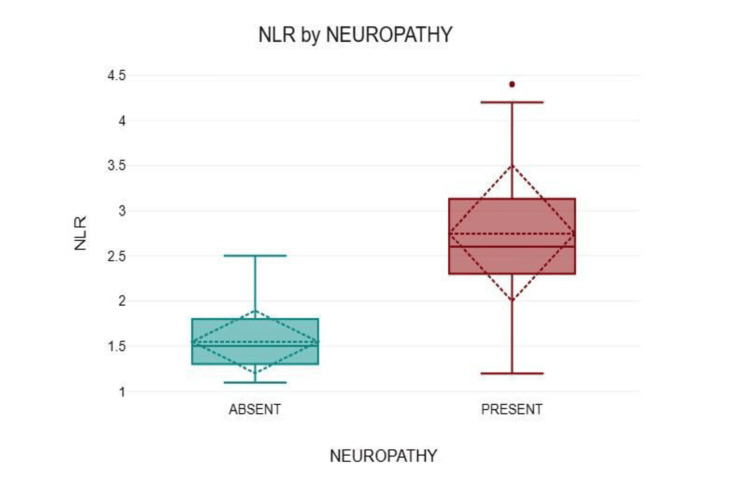
Comparison of the NLR between diabetes with neuropathy and without neuropathy NLR: Neutrophil-to-lymphocyte ratio

Participants without neuropathy (n = 77, 51.3%) had a mean NLR of 1.55 ± 0.35, while those with neuropathy (n = 73, 48.7%) had a mean NLR of 2.75 ± 0.76 (Figure 1).

**Figure 2:**
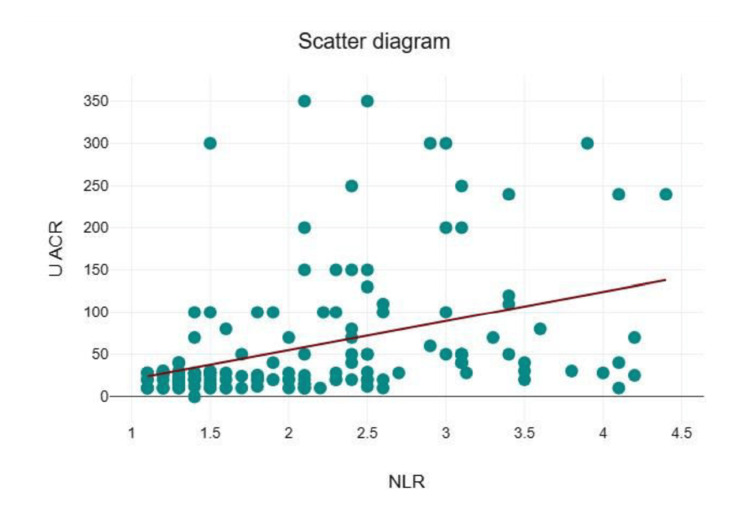
Correlation of the NLR and urine ACR (r = 0.39) NLR: Neutrophil-to-lymphocyte ratio; ACR: albumin-creatinine ratio

A strong positive correlation was observed between the NLR and neuropathy (r = 0.72, p < 0.001). Based on the urine ACR, participants were classified into three groups: <30, 30-300, and >300. The <30 group (n = 86, 57.3%) had a mean NLR of 1.79 ± 0.67, while the 30-300 group (n = 58, 38.7%) and >300 group (n = 6, 4%) had mean NLR values of 2.60 ± 0.83 and 2.65 ± 0.82, respectively. The NLR showed a moderate positive correlation with the urine ACR (r = 0.39, p < 0.001) (Figure 2).

**Figure 3:**
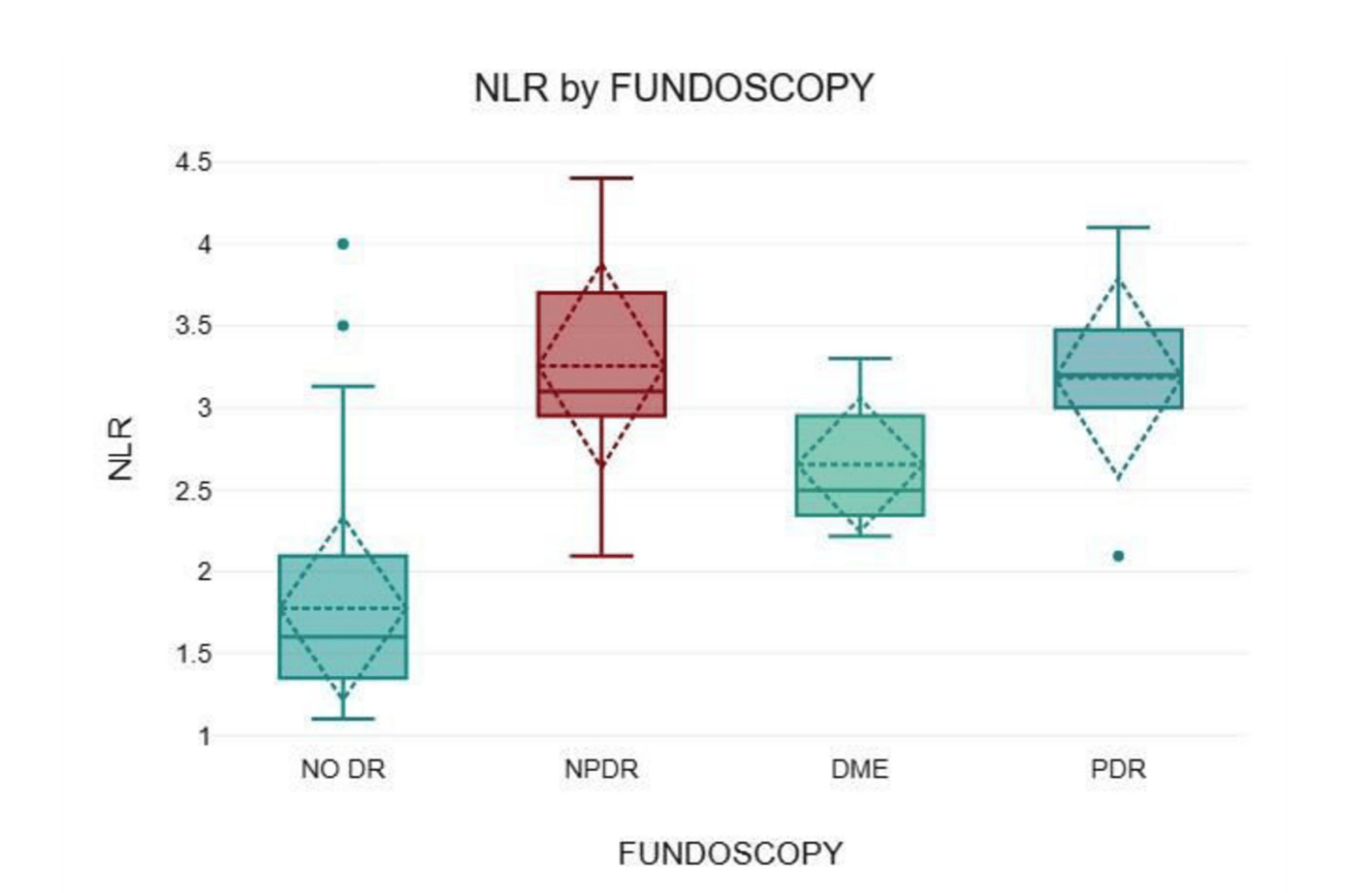
Comparison of the NLR across retinopathy categories NLR: Neutrophil-to-lymphocyte ratio; DR: diabetic retinopathy; NPDR: non-proliferative diabetic retinopathy; DME: diabetic macular edema; PDR: proliferative diabetic retinopathy

Diabetic retinopathy (DR) findings on fundoscopic examination were classified into four categories: no DR, non-proliferative DR (NPDR), proliferative DR (PDR), and diabetic macular oedema (DME). Most participants (74%, n=111) had no DR, with a mean NLR of 1.78 ± 0.56. NPDR, PDR, and DME were present in 18% (n=27), 4% (n=6), and 4% (n=6) of participants, respectively. The mean NLR values were 3.26 ± 0.64 in NPDR, 3.18 ± 0.67 in PDR, and 2.65 ± 0.44 in DME. Participants without retinopathy had a significantly lower mean NLR than those with NPDR, DME, or PDR (p < 0.001) (Figure 3).

The analysis revealed no significant relationship between NLR and factors such as age, sex, socioeconomic status, duration of diabetes, comorbid conditions, family history, history of addictions, or BMI.

Discriminatory ability of the NLR for microvascular complications

**Figure 4:**
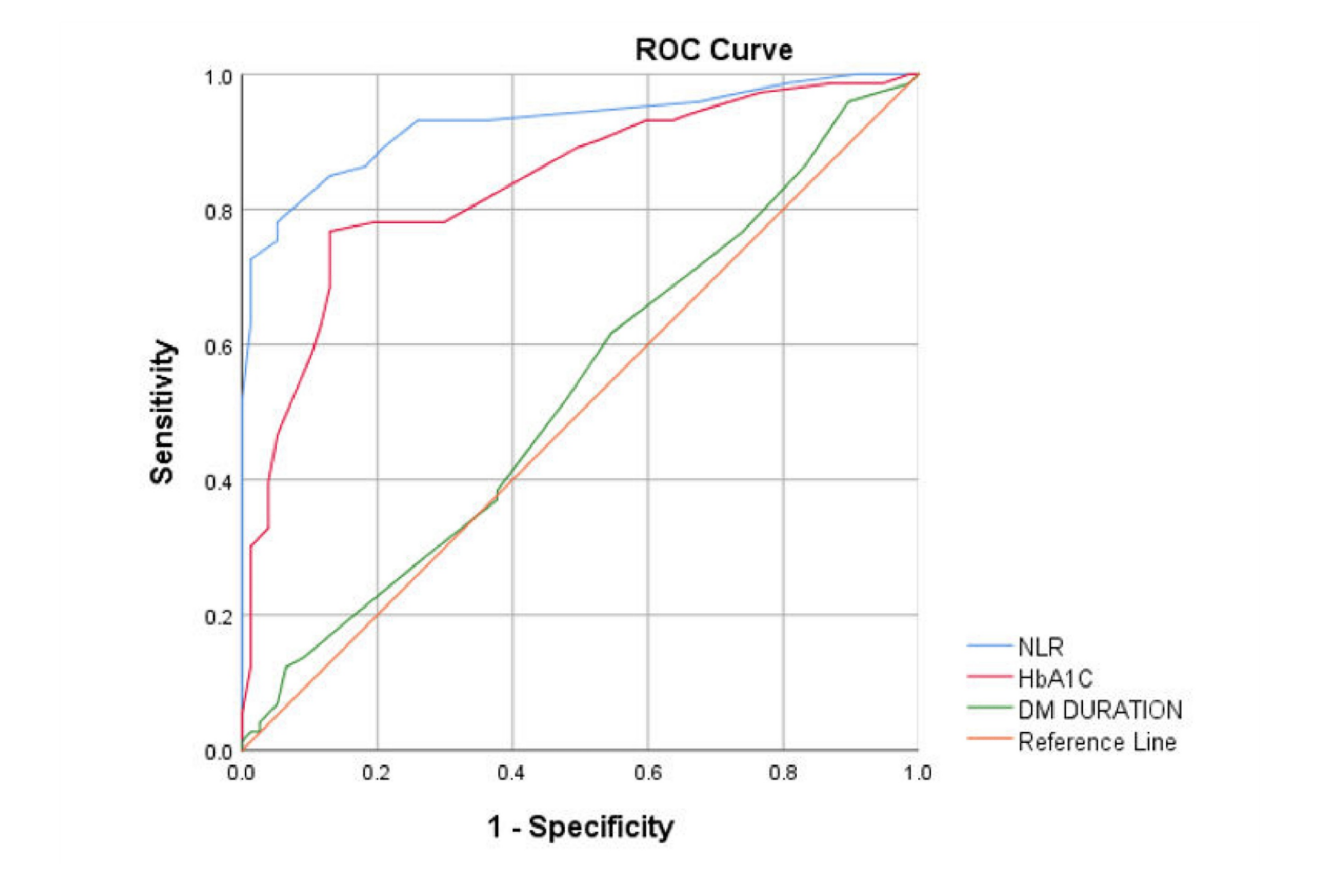
ROC curve analysis of the NLR for predicting neuropathy ROC: Receiver operating characteristic; NLR: neutrophil-to-lymphocyte ratio; DM: diabetes mellitus

ROC curve analysis was conducted to evaluate the ability of NLR, HbA1c, and disease duration to discriminate between patients with and without neuropathy, nephropathy, and retinopathy. For neuropathy, the NLR showed the highest AUC (0.926), followed by HbA1c (0.838) and duration of disease (0.533), indicating that the NLR is the strongest predictor (Figure 4).

**Figure 5:**
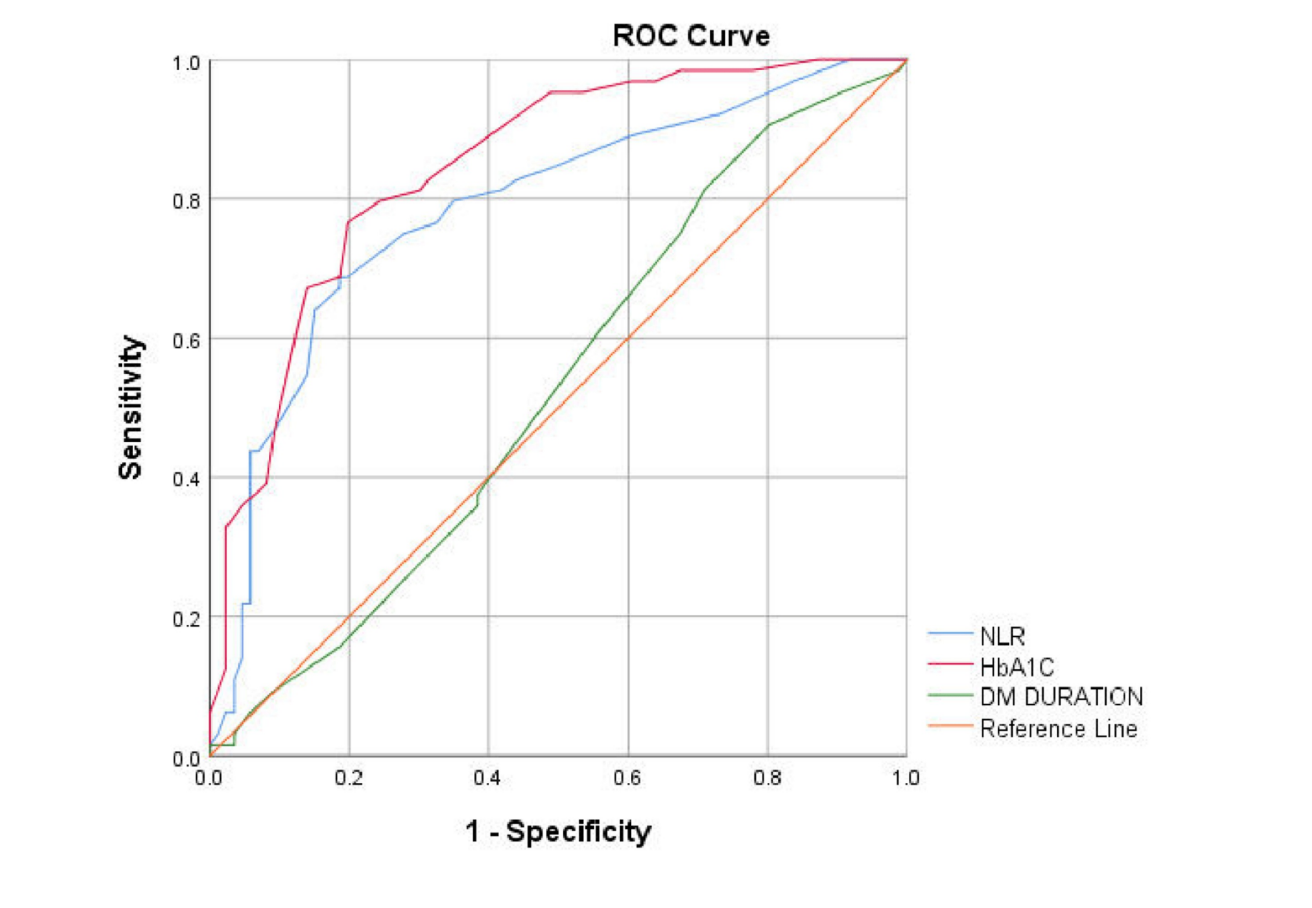
ROC curve analysis of the NLR for predicting nephropathy ROC: Receiver operating characteristic; NLR: neutrophil-to-lymphocyte ratio; DM: diabetes mellitus

An NLR cutoff of 1.75 provided a sensitivity of 93.2% and a specificity of 74% in detecting neuropathy. For nephropathy, HbA1c (AUC=0.845) had better discriminatory ability compared with the NLR (AUC=0.786) and duration of disease (AUC=0.527) (Figure 5).

**Figure 6:**
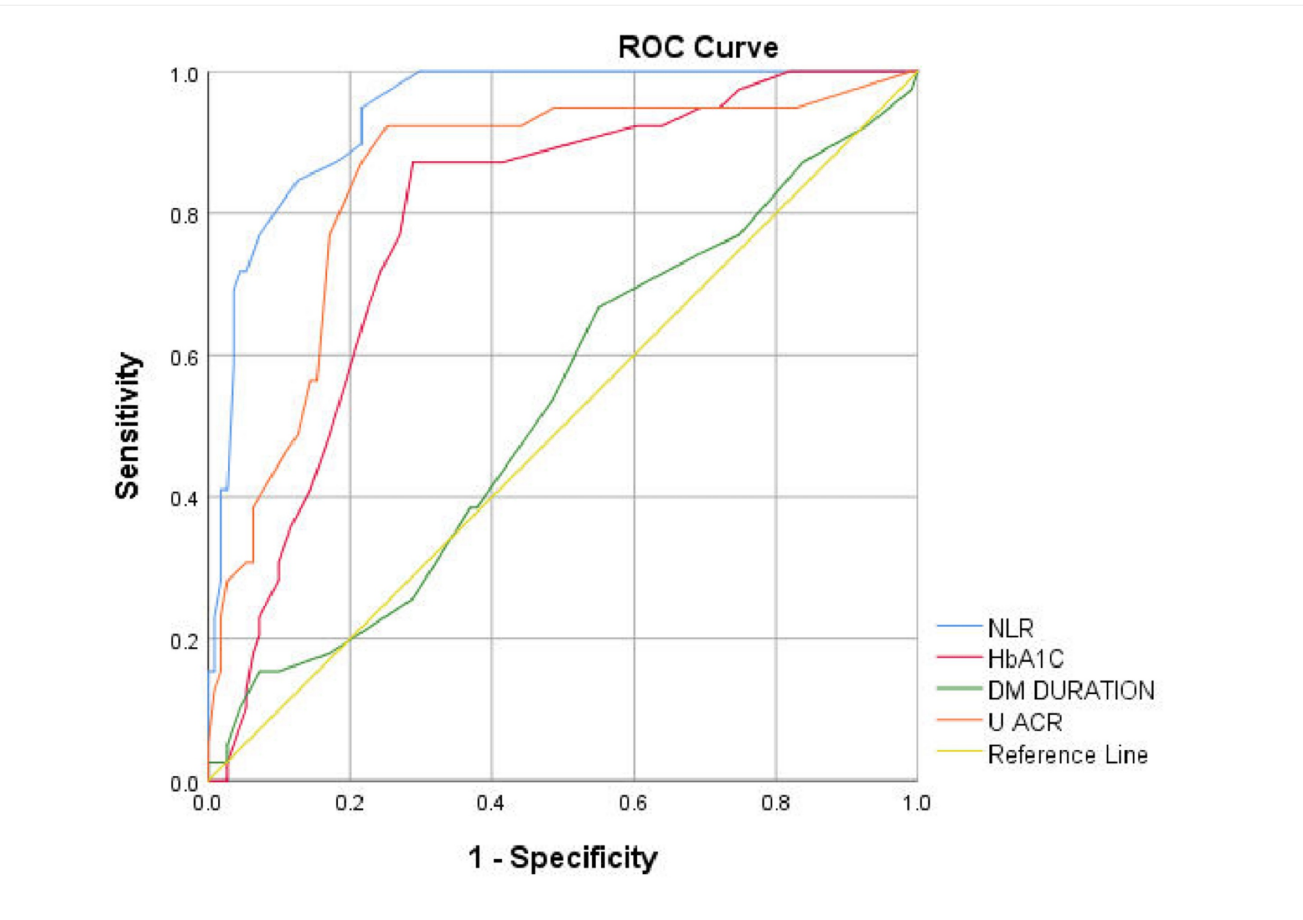
ROC curve analysis of the NLR for predicting nephropathy ROC: Receiver operating characteristic; NLR: neutrophil-to-lymphocyte ratio; DM: diabetes mellitus; ACR: albumin-to-creatinine ratio

For retinopathy, the NLR (AUC=0.942) showed the highest discrimination, outperforming the urine ACR (AUC=0.847), HbA1c (AUC=0.785), and duration of disease (AUC=0.531). With a cutoff value of 2.15, the NLR achieved a sensitivity of 94.9% and a specificity of 78.4% (Figure 6).

## Discussion

There is a growing need for low-cost, simple markers to predict diabetes-related end-organ damage. The participants had a mean age in their fifth decade, with a predominance of male subjects. These findings were comparable to previous Indian studies [4,7]. However, compared with studies by Ciray and Azab [8,9], our cohort included a higher proportion of male patients. This may be attributed to the inclusion of participants aged >40 years and to greater health-seeking behaviour among men. Such observations underscore the importance of increasing awareness of the nonspecific symptoms of diabetes among women to facilitate earlier diagnosis and timely treatment.

Most participants in our study had a family history of diabetes, which is consistent with observations from the EPIC-InterAct study [10], where 52% of 13,869 individuals reported a positive family history. More than three-fourths of our participants had comorbidities, with dyslipidaemia and systemic hypertension being the most common, both of which may contribute to elevated cardiovascular risk. Most participants (41.3%) were in the Obese I category. In our study, men had a higher WHR than women, consistent with previous reports [4]. An elevated WHR indicates truncal obesity, which is closely linked to both diabetes and cardiovascular disease, often referred to as "Syndrome X" [11].

T2DM is associated with chronic, persistent low-grade systemic inflammation, which may play a key role in the pathophysiology of hyperglycemia, insulin resistance, and diabetes-related vascular complications [12]. This persistent inflammatory state activates leukocytes (neutrophils and lymphocytes) and endothelial cells via a complex cytokine-mediated immunological cascade [13]. The NLR has emerged as a novel haematological marker of systemic inflammation, reflecting the dynamic interplay between innate immunity (neutrophils) and adaptive cellular immunity (lymphocytes). Evidence suggests that NLR is not only an indicator of inflammation but also holds potential as a predictive, diagnostic, and prognostic marker for secondary complications in T2DM [4,7,8,14,15].

In our study, the most common microvascular complication was diabetic neuropathy, followed by nephropathy and retinopathy. About one-fourth of participants had triopathy, while nearly half of the participants had no complication. Patients with neuropathy had a higher mean NLR than those without the condition, and the NLR showed a strong positive correlation with neuropathy (r = 0.72, p < 0.001). Our study demonstrated that the NLR has a much higher discriminative ability for detecting neuropathy compared with HbA1c, disease duration, and BMI. A study from China by Chen et al., involving 225 diabetic patients, similarly reported a significantly higher NLR in the neuropathy group, with a cut-off value of 2.485 and an AUC of 0.602 with a sensitivity of 38.00% and a specificity of 79.00% [16]. In a study by Lou et al., the NLR had a higher sensitivity and specificity (90% & 45%) than HbA1c at AUC 0.661 [17]. In a meta-analysis, it was observed that the NLR was a significant predictor in East Asia and India but not in Turkey and Egypt [18]. This correlation further supports the hypothesis that NLR contributes to the pathogenesis of chronic inflammatory diseases, even in sterile microenvironments, leading to microvascular complications [19].

We observed that elevated levels of proteinuria were associated with higher NLR. There was a positive correlation between the NLR and urine ACR (r = 0.39, p < 0.001). Similarly, Sachin et al., in 265 T2DM patients, found a significant positive correlation between the NLR and urine ACR (r = 0.33, p < 0.001) [4]. Also, a study by Chollangi et al. in 90 T2DM patients reported a similar positive correlation between the mean NLR and microalbuminuria (r=0.575) [7]. We found that the NLR had a lower discriminative ability than HbA1c for diagnosing nephropathy. In another study, it has been reported that an NLR threshold of 2.2 was able to predict diabetic nephropathy with a sensitivity and a specificity of 72.3% and 78.1%, respectively [20].

In our study, 1/4th of our participants had retinopathy. Patients with more advanced grades of retinopathy had higher NLR values. We demonstrated a significant correlation between the NLR and diabetic retinopathy (p<0.001). We also noticed that the NLR was a stronger predictor of retinopathy than the urine ACR. According to a meta-analysis of 14 studies encompassing 2,354 patients, Liu et al. reported that patients with diabetic retinopathy had significantly elevated NLR values compared to those without the condition (standardised mean difference = 0.77) [21]. Similarly, Yeter et al., in a study of 143 T2DM patients (including 53 with DME), found that NLR ≥ 2 (p < 0.001) was significantly associated with DME [22]. Several prior studies have reported no statistically significant difference in the NLR between patients with NPDR and those with PDR [21,23]. Although there are conflicting reports, our findings showed a positive correlation between NLR and diabetic retinopathy.

One of the main limitations of this study is its single-centre design and the relatively limited number of participants. Moreover, being cross-sectional, it could not assess changes in the NLR with the progression of microvascular complications in T2DM.

## Conclusions

The NLR showed a significant positive correlation with all microvascular complications in T2DM and outperformed other factors in predicting neuropathy and retinopathy. In resource-limited settings, it may serve as a cost-effective alternative marker. However, further large, prospective studies are needed to validate these results and determine their practical relevance in the management of T2DM.
